# Density of Sphingosine-1-Phosphate Receptors Is Altered in Cortical Nerve-Terminals of Insulin-Resistant Goto-Kakizaki Rats and Diet-Induced Obese Mice

**DOI:** 10.1007/s11064-023-04033-4

**Published:** 2023-10-04

**Authors:** Cecilia Skoug, Hüseyin Erdogan, Lotte Vanherle, João P. P. Vieira, Frank Matthes, Lena Eliasson, Anja Meissner, João M. N. Duarte

**Affiliations:** 1 Department of Experimental Medical Science (EMV), Faculty of Medicine, Lund University, Sölvegatan 19, BMC C11, 221 84 Lund, Sweden; 2 Wallenberg Center for Molecular Medicine, Lund University, Lund, Sweden; 3 Unit of Islet Cell Exocytosis, Department of Clinical Sciences Malmö, Lund University Diabetes Centre, Lund University, Malmö, Sweden; 4 Clinical Research Center, Skåne University Hospital, Malmö, Sweden; 5 Department of Physiology, Institute of Theoretical Medicine, Medical Faculty, University of Augsburg, Augsburg, Germany

**Keywords:** Synaptosomes, Sphingosine, Neuromodulation, Diabetes, Obesity

## Abstract

Sphingosine-1-phosphate (S1P) is a phosphosphingolipid with pleiotropic biological functions. S1P acts as an intracellular second messenger, as well as extracellular ligand to five G-protein coupled receptors (S1PR1-5). In the brain, S1P regulates neuronal proliferation, apoptosis, synaptic activity and neuroglia activation. Moreover, S1P metabolism alterations have been reported in neurodegenerative disorders. We have previously reported that S1PRs are present in nerve terminals, exhibiting distinct sub-synaptic localization and neuromodulation actions. Since type 2 diabetes (T2D) causes synaptic dysfunction, we hypothesized that S1P signaling is modified in nerve terminals. In this study, we determined the density of S1PRs in cortical synaptosomes from insulin-resistant Goto-Kakizaki (GK) rats and Wistar controls, and from mice fed a high-fat diet (HFD) and low-fat-fed controls. Relative to their controls, GK rats showed similar cortical S1P concentration despite higher S1P levels in plasma, yet lower density of S1PR1, S1PR2 and S1PR4 in nerve-terminal-enriched membranes. HFD-fed mice exhibited increased plasma and cortical concentrations of S1P, and decreased density of S1PR1 and S1PR4. These findings point towards altered S1P signaling in synapses of insulin resistance and diet-induced obesity models, suggesting a role of S1P signaling in T2D-associated synaptic dysfunction.

## Introduction

Bioactive lipids are important for brain function. In particular, the homeostasis of the sphingolipids ceramide-1-phosphate, sphingosine, and sphingosine-1-phosphate (S1P) has been described to regulate proliferation, differentiation, cell growth and inflammation in the cells of the central nervous system (CNS) [1]. S1P is produced from ceramide by ceramidase followed by phosphorylation by sphingosine kinase (SphK1/2) [2]. S1P is abundantly produced by erythrocytes, platelets and endothelial cells [3, 4]. Although, S1P synthesis in the CNS has also been reported, namely by astrocytes after fibroblast growth factor stimulation [5].

S1P in the extracellular space binds to its five specific G-coupled receptors S1PR1-5, which signal through diverse downstream pathways [1]. All S1P receptors are expressed in the CNS [6]. They participate in multiple important functions during CNS development, and further contribute to the development and/or resolution of neurodegeneration in pathological conditions such as ischemic stroke [7], multiple sclerosis [8], hearing loss [9] and seizures [10, 11]. S1P receptors have received attention in the field of multiple sclerosis, for which immunomodulation is achieved by the oral drug Fingolimod (FTY720) that activates S1PR1,3–5 [12]. S1PR1 is the most studied S1P receptor with important roles in angiogenesis and neurogenesis [13]. S1PR2 is a modulator of neuronal excitability during neuronal development [14] and controls spontaneous activity of cultured neurons [6]. S1PR3 receptor controls activity of microglia and their participation in neuroinflammation [15–17], and is highly expressed in astrocytes, where it regulates astrogliosis via activation of the small GTPase RhoA [18]. Until recently, S1PR4 has received little attention in the CNS [19]. Our recent work proposed S1PR4 presence in synapses, and a role in neuronal activity control [6]. Furthermore, previous studies have demonstrated loss of sphingosine kinase activity and lowering of brain region-specific S1P levels early in the progression of Alzheimer’s disease [20, 21].

Recent evidence also suggests an important role for S1P and its generating enzymes SphK1/2 in the development of diabetes [22] through adverse effects on endothelial function [17, 23], hepatic insulin sensitivity and secretion [24], regulating insulin secretion in pancreatic β-cell [25], and contributing to diabetes-associated inflammation [23].

The sphingosine rheostat has been extensively studied in diabetes and insulin resistance, with special focus on ceramide and ceramide-1-phosphate [22]. Because S1P is involved in a plethora of cellular functions, a tight regulation of S1P homeostasis seems important for proper brain functioning, which would be disrupted in metabolic syndrome and diabetes. Diabetes impacts synapses with negative consequences for brain function, including memory performance [26]. Furthermore, rodent models of diabetes show altered neuromodulation systems operated by adenosine [27–29], ATP [30], or endocannabinoids [31, 32], which control synaptic activity and brain energy metabolism, and might interact with brain insulin signaling [33]. These systems can also afford neuroprotection. For example, pharmacologically targeting the altered adenosinergic system components confers neuroprotection and improves memory performance in diabetes models [28, 29, 34]. It is hitherto unknown whether the neuromodulation system operated by S1P in the CNS is altered in diabetes and metabolic syndrome.

In this study, we set out to test the hypothesis that T2D impacts the neuromodulation system operated by S1P in the CNS. Since S1PRs are present in nerve terminals of the cortex [6], we determined changes induced by T2D in the density of S1PR1-4 in cortical synaptosomes from insulin-resistant Goto-Kakizaki (GK) rats and from diet-induced obese mice, which are models with well-established brain dysfunction (e.g., [29, 35, 36]).

## Methods

### Animals

**Fig. 1:**
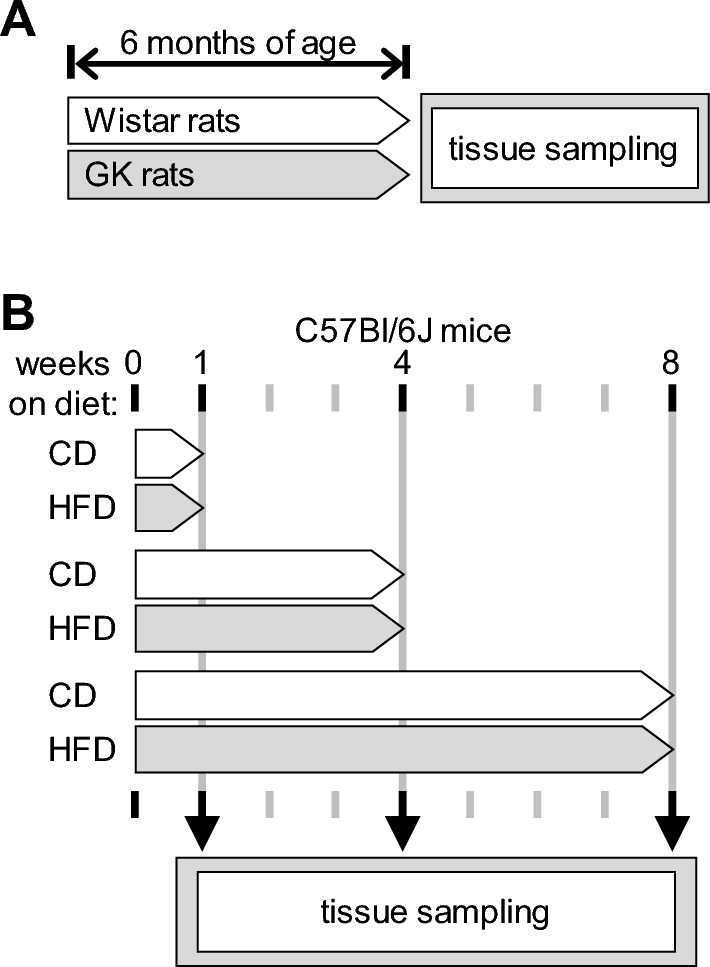
Study design. Samples from Wistar and GK rats were collected at 6 months of age (**A**). Mice were kept on CD or HFD for 1, 4 or 8 weeks before tissue sampling (**B**)

Experiments were performed according to EU Directive 2010/63/EU under approval of the Malmö/Lund Committee for Animal Experiment Ethics (permit numbers 994/2018 and 9987/2020) and are reported following the ARRIVE guidelines (Animal Research: Reporting In Vivo Experiments, NC3Rs initiative, UK). Male and female GK rats were obtained from a local colony, and male and female age-matched Wistar rats from Janvier (Saint Berthevin, France) were used as controls [29]. Wistar rats were housed in the facility for at least a month before experimentation. Eight weeks-old C57BL/6 J mice were purchased from Taconic (Ry, Denmark). Only male mice were used due to sex differences in responses to diet-induced obesity (see [35], and references therein). Animals were housed in ventilated cages enriched with a cylinder, wood toys and nesting material, at controlled temperature of 22 °C, 50–60% humidity and a 12:12-h light–dark cycle. Food and water were provided ad libitum. Rats were kept on a regular chow and were euthanized at 6 months of age (Fig. 1A). Mice were fed a lard-based high-fat diet (HFD; 60% calories from fat) or a control diet (CD, 10% calories from fat) from Research diets (New Brunswick, NJ-USA), as previously described [35]. Mice were held on the diet for 1 week, 1 or 2 months, starting from 9 weeks of age (Fig. 1B).

### Glucose Tolerance Test and Insulin Determination

A glucose tolerance test (GTT) was performed 1–3 days before sacrifice. Food was removed at 08:00 for 6 h, and mice were put into clean cages to avoid coprophagy before and during the test. Before each test, a blood sample was collected from vena saphena into a heparinized tube for determination of plasma insulin concentration. Glycemia was measured from tail tip blood with the Accu-Chek Aviva glucometer (Roche, Solna, Sweden). Mice were then administered 2 g/kg glucose i.p. from a 20%(w/v) solution in saline, followed by determination of glucose levels after 15, 30, 60, 90 and 120 min.

Plasma insulin was measured with ELISA kits from Mercodia (Uppsala, Sweden; #10-1250-01 for rats; #10-1247-10 for mice).

### Preparation of Nerve Terminal-Enriched Membranes

After weighing and measuring tail-tip blood glucose, animals were anesthetized with isoflurane and quickly decapitated. Trunk blood was collected, and plasma was stored at − 80 °C for insulin and S1P determination. Brains were quickly dissected, frozen in N_2_ (l) and stored at − 80 °C until further experiments. Synaptosomes were prepared as described previously [36]. Briefly, cortical tissue was homogenized (12 strokes at 800 rotations/min) with a glass-teflon Potter–Elvehjem homogenizer (rotor head InterMed/STIR20) in Sucrose-HEPES buffer (0.32 mol/L sucrose, 1 mmol/L EDTA, 10 mmol/L HEPES, 1 mg/mL bovine serum albumin, pH 7.4) at 4 °C. Homogenates were centrifuged at 3000 *g* for 10 min at 4 °C (Beckman Coulter/Avanti J-20 XP). After, supernatants were centrifuged at 14,000 *g* for 12 min at 4 °C. The pellet was re-suspended in 1 mL of 45% (v/v) Percoll (GE Healthcare, Uppsala, Sweden) solution prepared in Krebs-HEPES buffer (in mmol/L: 140 NaCl, 5 KCl, 10 HEPES, 1 EDTA, 5 glucose, pH 7.4), and then centrifuged at 21,000 *g* for 2 min at 4 °C. The top layer (rich in synaptosomes) was washed by re-suspending in Krebs-HEPES buffer and centrifuging again. The resulting pellets were re-suspended in Krebs-HEPES solution containing protease inhibitors (cOmplete cocktail from Roche, Mannheim, Germany), and stored at − 80 °C until immunoblotting.

### Immunoblotting

Total protein content of the samples was measured with the bicinchoninic acid assay (kit from Pierce, ThermoFisher Scientific, Uppsala, Sweden). Then, Western blotting was carried out as detailed by Lizarbe et al. [36]. Briefly, samples were dissolved in sample buffer (#NP0007, Invitrogen, ThermoFisher), boiled at 95 °C for 5 min, and then separated by SDS-PAGE in 4–12% Bis–Tris gradient gels (#NP0336, Invitrogen), followed by transfer onto nitrocellulose membranes of 0.45-μm pore size (#GE10600002, GE Healthcare). The membranes were blocked for 1–2 h in Tris-buffered saline (in mmol/L: 20 Tris, 150 NaCl, pH 7.6) containing 5% (w/v) skim milk, 1% (v/v) Tween-20, and then sequentially incubated with primary and secondary antibodies (Table 1) diluted in this blocking solution. Immunoblots were developed with the chemiluminescence Super-Signal kit (#34,580, ThermoFisher). Whenever necessary, sensitivity was enhanced with the biotin-streptavidin kit VectaStain ABC-HRP according to manufacturer’s instructions (#PK-4000, Vectorlabs, CA-USA). Luminescence was detected using the Chemidoc XRS + interfaced to Image Lab 5.2.1 (Biorad, Stockholm, Sweden).

**Table 1:** Antibodies used for Western blot (WB) and molecular weight of the analyzed band

Antibody	Dilution	Source	Molecular weight (kDa)
Rabbit anti-S1PR1	1:1,000	ThermoFisher (PA1-1040)	47 kDa
Rabbit anti-S1PR2	1:500	Origene (AP01311PU-N)	42 kDa
Rabbit anti-S1PR3	1:1,000	Origene (TA329055)	50 kDa
Rabbit anti-S1PR4	1:1,000	Novus (NBP2-24,500)	39 kDa
Rabbit anti-SNAP25	1:5,000	Abcam (ab109105)	25 kDa
HRP-tagged anti-β-actin	1:10,000	Sigma-Aldrich (A3854)	42 kDa
HRP-tagged anti-rabbit IgG	1:5,000	Abcam (ab6802)	–
Biotinylated anti-rabbit IgG	1:5,000	Vectorlabs (BA-1000)	–

### Mass Spectrometry

Plasma samples obtained from trunk blood, and cortical homogenates in phosphate-buffered saline (PBS; in mmol/L: 137 NaCl, 2.7 KCl, 1.5 KH_2_PO_4_, 8.1 Na_2_HPO_4_, pH 7.4) were spiked with deuterated S1P as internal standard (S1P-D7 > 99% deuterated; Avanti Polar Lipids/Merck, Darmstadt, Germany), and S1P was extracted as described in [37]. Extracts were dried under a nitrogen stream, dissolved in methanol, and subjected to liquid chromatography-coupled tandem mass spectrometry as previously described [38, 39].

### Statistics

Results are shown as mean ± SD, and were analyzed with Prism 9.4.1 (GraphPad Software, San Diego, CA). Normal distribution was assessed with the Kolmogorov–Smirnov test. In the presence of normality deviations, results were analyzed using Mann–Whitney test or Kruskal–Wallis test followed by Dunn’s multiple comparisons. Normally distributed data was analyzed with unpaired, two-tailed Student *t*-test or ANOVA followed by independent comparisons with the Fisher’s least significant difference (LSD) test. Significance was accepted for *P* < 0.05.

## Results

We have previously detailed metabolic phenotypes of insulin-resistant GK rats [29] and HFD-induced obese mice [40]. In the present study, we have confirmed that GK rats displayed lower body weight, increased glycemia, and similar fed plasma insulin concentration, when compared to controls (Table 2). HFD-fed mice showed increased body weight, a tendency for increased fasting glycemia and plasma insulin concentrations, reduced glucose tolerance, and increased insulin resistance, when compared to controls (Table 3).

**Table 2:** Metabolic parameters of GK and Wistar rats

	Wistar (*n* = 3)	GK (*n* = 3)		F(DFn,DFd), *P*-value
Body weight (g)				Interaction F(1,8) = 38, *P* < 0.001
Male	582 ± 37	353 ± 44	*P* < 0.001	Diabetes F(1,8) = 47, *P* < 0.001
Female	279 ± 7	266 ± 20	n.s.	Sex F(1,8) = 122, *P* < 0.001
Glycemia (mmol/L)				Interaction F(1,8) = 18, *P* = 0.003
Male	6.5 ± 0.8	18.2 ± 3.4	*P* < 0.001	Diabetes F(1,8) = 39, *P* < 0.001
Female	6.3 ± 0.6	8.7 ± 1.1	*P* = 0.035	Sex F(1,8) = 19, *P* = 0.002
Plasma insulin (µg/L)				Interaction F(1,8) = 0.096, n.s.
Male	2.0 ± 0.6	2.8 ± 1.1	n.s.	Diabetes F(1,8) = 3.6, n.s.
Female	0.9 ± 0.3	2.0 ± 1.1	n.s.	Sex F(1,8) = 4.0, n.s.

Data is mean ± SD (*n.s.* non-significant difference, *P* > 0.05)

**Table 3:** Metabolic parameters of HFD-fed mice compared to CD-fed mice

	CD (*n* = 9–16)	HFD (*n* = 10–16)		F(DFn,DFd), *P*-value
Body weight (g)				
1 week	23.6 ± 1.4	26.0 ± 1.8	*P* < 0.001	Interaction F(2,70) = 2.2, n.s.
1 month	28.3 ± 1.5	31.9 ± 2.1	*P* < 0.001	Diet F(1,70) = 50, *P* < 0.001
2 months	30.6 ± 2.3	35.2 ± 3.4	*P* = 0.017	Time F(2,70) = 91, *P* < 0.001
Fasting glycemia (mmol/L)				
1 week	8.6 ± 1.3	10.0 ± 0.9	n.s.	Interaction F(2,70) = 1.67, n.s.
1 month	8.2 ± 1.5	11.0 ± 3.0	*P* < 0.001	Diet F(1,70) = 17, *P* < 0.001
2 months	8.4 ± 1.1	9.2 ± 1.1	n.s.	Time F(2,70) = 1.9, n.s.
Fasting plasma insulin (µg/L)				
1 week	1.7 ± 1.1	1.8 ± 0.8	n.s.	Interaction F(2,62) = 0.85, n.s.
1 month	0.6 ± 0.3	1.0 ± 0.6	n.s.	Diet F(1,70) = 95, *P* = 0.023
2 months	0.8 ± 0.7	1.5 ± 0.8	*P* = 0.026	Time F(2,70) = 10, *P* < 0.001
2-h glycemia in GTT (mmol/L)				
1 week	8.3 ± 1.5	12.0 ± 1.8	*P* < 0.001	Interaction F(2,70) = 0.66, n.s.
1 month	7.7 ± 1.0	11.5 ± 2.2	*P* < 0.001	Diet F(1,70) = 95, *P* < 0.001
2 months	7.3 ± 1.1	12.1 ± 2.5	*P* < 0.001	Time F(2,70) = 0.76, n.s.
HOMA-IR*				
1 week	13.8 ± 9.2	17.7 ± 7.3	n.s.	Interaction F(2,62) = 0.42, n.s.
1 month	4.7 ± 2.4	11.2 ± 7.4	*P* = 0.011	Diet F(1,62) = 12, *P* < 0.001
2 months	6.7 ± 6.2	14.8 ± 9.1	*P* = 0.011	Time F(2,62) = 6.8, *P* = 0.002

Data is mean ± SD (*n.s.* non-significant difference, *P* > 0.05)

*Homeostatic model assessment for insulin resistance calculated from fasting insulinemia and glycemia

**Fig. 2:**
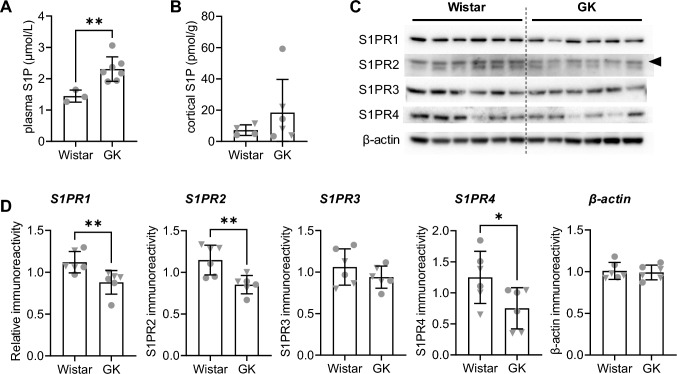
S1P concentrations and cortical nerve-terminal density of S1PRs of GK and Wistar rats. S1P concentration in **A** plasma and **B** cortical homogenates. **C** Immunoblots for S1PR1-4 and β-actin (protein loading control) after SDS-PAGE separation of 15 µg of protein from nerve terminal-enriched cortical membranes. **D** relative immunoreactivity estimated from the relative signal intensity in the immunoblots. Data shown as mean ± SD overlaid on individual data points (*n* = 3–7; circles = male rats, triangles = female rats). Symbols over data-points indicate significant differences between Wistar and GK rats (*P < 0.05, **P < 0.01) based on Mann–Whitney (for cortical S1P concentration and S1PR1 immunoreactivity) or Student t-tests (remaining comparisons)

Plasma concentrations of S1P were higher in GK than Wistar rats (+ 60%, *P* = 0.008, Fig. 1A). However, S1P levels in homogenates from the cortex of GK rats were not significantly different from those in Wistar rats (*P* > 0.05, Fig. 2B). Nerve terminal-enriched membranes from GK rats showed lower immunoreactivity of S1PR1 (− 21%, *P* = 0.004), S1PR2 (− 26%, *P* = 0.006) and S1PR4 (− 40%, *P* = 0.046,) than those from control Wistar rats (Fig. 2C–D). S1PR3 immunoreactivity was similar between the groups (*P* > 0.05).

**Fig. 3:**
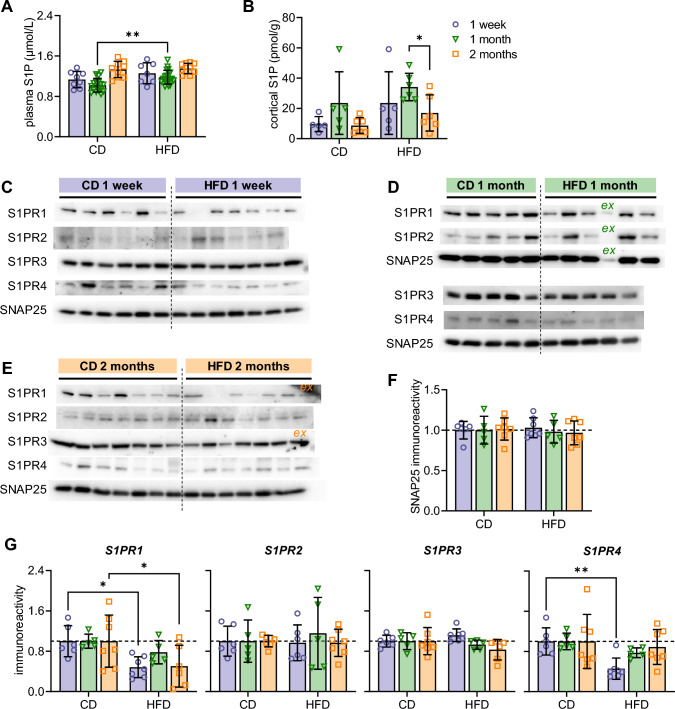
S1P concentrations and cortical nerve-terminal density of S1PRs of mice fed HFD and CD. S1P concentration in **A** plasma and **B** cortical homogenates. **C** Immunoblots for S1PR1-4 and SNAP25 (protein loading control) after SDS-PAGE separation of 15 µg of protein from nerve terminal-enriched cortical membranes. **D–G** Scatter plots show relative immunoreactivity estimated from the signal intensity in the immunoblots after normalization to mean of CD samples. Data shown as mean ± SD overlaid on individual data points (*n* = 5–20). Circles, triangles, and squares represent 1 week, 1 month, and 2 months of diet intervention, respectively. Symbols over data-points (*P < 0.05, **P < 0.01) indicate significant mean differences from post-tests comparing CD vs HFD within each age, or age within each diet, using Kruskal–Wallis test followed by Dunn’s multiple comparisons (in panel B), or ANOVA followed by independent comparisons with the Fisher’s least significant difference (LSD) test (in panels A, F–G). Samples indicated by *ex* were excluded from analysis

Plasma concentrations of S1P were increased by both HFD exposure and age (interaction F(2,64) = 1.4, *P* > 0.05; diet F(1,64) = 7.2, *P* = 0.009; time F(2,64) = 17, *P* < 0.001). In particular, HFD-feeding increased plasma S1P levels by 11–16%, most prominently at 1 month after diet intervention (Fig. 3A). In the cortex, S1P levels were also increased with HFD, and showed a variation with age that was unrelated to plasma S1P levels (interaction F(2,27) = 0.12, *P* > 0.05; diet F(1,27) = 5.5, *P* = 0.026; time F(2,27) = 4.4, *P* = 0.022, Fig. 3B). The density of S1PRs was analyzed in nerve-terminal membranes from the cortex of mice fed a CD or HFD for 1 week, 1 month, and 2 months (Fig. 3C–E). SNAP25 analyzed as constitutive protein showed no HFD-induced changes (Fig. 3F). Relative to CD, HFD exposure reduced S1PR1 immunoreactivity (interaction F(2,30) = 0.64, *P* > 0.05; diet F(1,30) = 13, *P* = 0.001; time F(2,30) = 0.64, *P* > 0.05; Fig. 3G), which is particularly prominent at 1 week (− 52%, *P* = 0.011) and 2 months of HFD feeding (− 50%, *P* = 0.014). The density of S1PR2 and S1PR3 was not modified by HFD feeding (Fig. 3G). S1PR4 immunoreactivity in cortical nerve terminal membranes was lower in HFD-fed mice than controls (interaction F(2,31) = 1.6, *P* > 0.05; diet F(1,31) = 7.4, *P* = 0.011; time F(2,31) = 1.6, *P* > 0.05; Fig. 3G), most prominently after 1 week of HFD feeding (− 54%, *P* = 0.005), and recovering to control levels at 2 months of HFD.

## Discussion

This study demonstrates for the first time that S1PR density in cortical nerve terminals is reduced in lean and obese models of insulin resistance, along with possible increases of S1P concentrations in the cortex, implying a role for S1PR signaling in the neuropathological process that occurs in T2D. Since S1PR activation generally attenuates neuronal activity [6], lower S1PR density might lead to overall cortical hyper-excitability.

From those analyzed, S1PR1 is the receptor with the highest expression level in the CNS. Its density was decreased in cortical synapses of both insulin-resistant GK rats and HFD-fed mice, relative to their respective controls. Previous studies have reported similar S1PR1 level reductions in the hypothalamus of HFD-fed rats or mice, as well as in the leptin deficient ob/ob mice [41]. Silva et al. proposed that S1P is an appetite inhibitor through S1PR1 signaling, and reduced S1PR1 levels in the hypothalamus of obese models were associated with increased food intake. S1PR1 activation inhibits neuronal activity [6] and, therefore, the S1PR1 reduction observed in cortical synapses is likely to result in loss of S1P-dependent synaptic control. However, S1PR1 is ubiquitously expressed, and exert a multitude of regulatory actions, not being specific to synapses. Thus, a potential approach of pharmacologically targeting S1PR1 for synaptic modulation might also trigger a plethora of non-synaptic actions. Adding to the complexity of S1PR1 signaling, others have reported that specific S1PR1 activation increases excitability of some sensory neurons in the rat dorsal root ganglia [42]. Given the ubiquitous actions of S1P, any systemic interventions on S1PR1 should be taken with caution.

The density of both S1PR2 and S1PR4 was decreased in cortical synapses from GK rats compared to controls. Considering the role of S1PR2 as neuromodulator in excitatory neurons [14], and its preferential localization at the pre-synaptic level in the cortex [6], dampening of S1PR2 signaling in GK rats is likely to contribute to the synaptic dysfunction and impaired synaptic plasticity [29]. Our previous study [6] showed that specifically S1PR2 and S1PR4 activation results in dampening of spontaneous spiking frequency of cultured primary neurons. A reduction in levels of synaptic S1PR2/4 could thus result in uncontrolled, excessive synaptic activity that might lead to excitotoxicity and synaptic damage [26]. We thus speculate that S1PR2/4 activation might allow for synaptic protection in diabetic conditions.

HFD-fed mice did not reproduce the T2D-induced S1PR2 alterations observed in GK rats. Moreover, S1PR4 density was reduced in short- but not long-term HFD, when compared to the respective controls. Besides obesity, a key difference between the two models is the hyperglycemia in GK rats [43, 44] that is negligible in HFD-fed mice [26, 36]. On the other hand, both models become insulin resistant. In this regard, it needs to be noted that S1P signaling through S1PR2 has been shown to interact with insulin signaling, and might participate in the development of insulin resistance in peripheral cells [45]. Given the ability of S1PR2 to inhibit insulin-mediated signals, it is plausible that reduced S1PR2 in synaptic membranes results in enhanced insulin signaling. Thus, S1PR2 might contribute to the development of central insulin resistance. In turn, putatively increased S1PR2 signaling due to higher S1P concentrations in HFD-fed mice could also dampen synaptic insulin signaling. Loss of tonic insulin receptor signaling in nerve terminals is believed to contribute to memory impairment [33, 46].

Although S1PR5 is present in the CNS, its relevance for neuromodulation at the synaptic level remains to be established ([6], and references therein). Therefore, we have not investigated diabetes-induced alterations of S1PR5 density in the present study.

Concentrations of S1P measured by ELISA were found to increase in the liver of patients with T2D as well as in streptozotocin-induced T2D rats [47]. Plasma S1P concentration also increases in the ob/ob mouse model, mice exposed to HFD for 6 weeks, and in a population of young obese humans [48]. Interestingly, plasma S1P in humans was found to be associated with body fat, insulin levels, insulin resistance (by HOMA-IR), as well with cholesterol [48]. The same study reported an increase in concentrations of S1P in plasma after 12 h of fasting, suggesting a relation between S1P levels and increased inter-organ lipid flux. In our study, plasma samples for S1P determination were collected under fed state, which we therefore expect to depict diabetes-induced S1P changes rather than acute fasting-induced mobilization of lipid stores.

While plasma S1P levels increase in non-diabetic young adults with obesity (< 30 years old; body mass index ~ 37 kg/m^2^) relative to lean controls [48], others have reported that T2D is associated with a decrease of plasma S1P concentrations [49, 50]. Namely, these studies found lower plasma S1P concentrations in individuals with T2D relative to healthy controls matched for age and body mass index (average: ~ 60 years old and ~ 29 kg/m^2^ in Vaisar et al. [49]; 44–49 years old and 25–26 kg/m^2^ in Sui et al. [50]). While reported effects of obesity and T2D on plasma S1P levels are opposite, these studies differ in the studied populations, age of the subjects, and methods for S1P extraction and detection.

S1P levels in the cortex are reported to be lower than in other brain regions [51], and we now found S1P concentrations increased in the cortex of HFD-fed mice. To our knowledge, this is the first study investigating S1P concentration in the cerebral cortex of T2D/pre-diabetic animal models. We report an HFD-induced increase of S1P in cortical tissue that seems to be independent of HFD-induced increases in circulating S1P levels. In turn, in GK rats, the large variance of S1P concentrations measured in the cortex precluded determining the expected diabetes-induced increase in S1P levels. This contrasts the concurrent increases of brain and plasma S1P that have previously been reported in a mouse model of hypertension [52].

The assessment of S1PR density in mouse cortical synapses across the HFD treatment is limited to the effects of the exposure to the diet, and not the effect of age. This is due to the semi-quantitative nature of immunoblotting methods, and the fact that we have decided to analyze all age-matched samples in parallel. Nevertheless, mice were treated for a relatively short period of time (only 2 months), at an age range from about 2 to 4 months, during which changes of S1PR density might not be as important as effects of the diet. Another limitation of this study is that despite including both sexes in the GK rat part of the study, analysis of plasma S1P levels was only available for males. We are not able to determine sex effects on plasma S1P concentrations, although they were not apparent in cortical levels of S1P. Finally, another important limitation of this study is that the nerve-terminal enriched membranes obtained with our protocol also contain non-synaptic components, such as the perisynaptic astrocytic processes. In particular, S1PR1 is abundant in astrocytes, and the observed S1PR1 reduction could be due to alterations in astrocytic rather than synaptic compartments. However, in total membranes obtained as previously described [27], we have not observed any S1PR1 reduction in GK vs Wistar rats, or HFD-fed mice vs controls (data not shown). Thus, S1PR density alterations in the present study most likely take place within the synaptic membranes.

In conclusion, we have observed a decrease in the protein levels of three S1P receptors in the cortex of the GK model that were likely unrelated to cortical concentrations of S1P. Two of the altered receptors, namely S1PR2 and S1PR4, have been proposed to control neuronal activity and synaptic transmission ([6], and references therein). Furthermore, S1PR4 levels were also decreased in the cortex of a diet-induced obese mouse model with metabolic syndrome, along with increased cortical levels of S1P. Altogether, these results point towards T2D-induced alterations of the neuromodulation system to which S1P signaling contributes. Our results open the door for testing brain-specific S1P-S1PR modulation as a potential neuroprotection strategy in diabetes.

## Data Availability

The datasets from the current study are available from the corresponding author on reasonable request.

## Abbreviations

CNS: Central nervous system
HFD: High-fat diet
GK: Goto-Kakizaki
GTT: Glucose tolerance test
S1P: Sphingosine 1-phosphate
S1PR: Sphingosine-1-phosphate receptors
Sph: Sphingosine
SphK: Sphingosine kinase
T2D: Type 2 diabetes
